# Beyond the hippocampus: outstanding academic achievements despite significant memory impairments. A case report on temporal epilepsy

**DOI:** 10.1192/j.eurpsy.2026.11062

**Published:** 2026-07-31

**Authors:** L. C. Rocha-Reza, M. Larrayoz-Iribarren, E. Fernández-Jiménez

**Affiliations:** 1 Servicios de Atención Psiquiátrica, Hospital Psiquiátrico Fray Bernardino Álvarez, Ciudad de México, Mexico; 2 Servicio Navarro de Salud-Osasunbidea, Pamplona; 3 Department of Child and Adolescent Psychiatry, La Paz University Hospital; 4 Hospital La Paz Institute for Health Research (IdiPAZ), Madrid; 5 Faculty of Law, Education and Humanities, Universidad Europea de Madrid, Villaviciosa de Odón (Madrid), Spain

## Abstract

**Introduction:**

Mesial temporal structures, particularly the hippocampus, play an important role in memory formation and retrieval. Therefore, if there is hippocampal injury, there is supposed to be a significant dysfunction in daily activities and/or academic/work performance that requires a greater memory load. Also, if this brain damage occurs in the early stages of neurodevelopment, it may result in significant deviations from the expected milestones compared to the reference age group.

**Objectives:**

To present the neuropsychological findings and academic performance of a child with temporal epilepsy and bitemporal mesial sclerosis.

**Methods:**

This case report included the patient´s clinical history, brain MRI and neuropsychological assessment at two measurement points with a 24-month interval. The main outcomes of neuropsychological testing included the Spain-Complutense Verbal Learning Test for Children (TAVECI), which is a Spanish test based on the California Verbal Learning Test; the Rey-Osterrieth Complex Figure (ROCF); the *Vocabulary* subtest of the Wechsler Intelligence Scale for Children, fifth edition (WISC-V); and the *Digit Span* subtest of the WISC-V.

**Results:**

An 8 years-old female patient, with bitemporal mesial sclerosis and temporal epilepsy, which was diagnosed when she was 6 years old. Neuroimaging revealed anatomical damage to the mesial temporal structures in both lobes. The results of the neuropsychological assessments indicated significant memory impairments longitudinally. In particular, short- and long-term impairments were observed in both visuo-constructive and verbal memory. However, improvement was identified in verbal recognition, although she still had a very low performance. In contrast, auditory working memory levels were stable and within the normotypic range. Interestingly, improvements were observed in semantic memory, reaching slightly higher-than-normal levels. Despite these indicators, the patient had outstanding academic achievements.

**Conclusions:**

Temporal epilepsy is strongly associated with mesial temporal sclerosis and memory impairments, which have important dysfunctions in daily activities and other functional domains. In this patient, the neuropsychological and neuroimaging (hippocampal damage) findings converge, in contrast to the fact that this child has outstanding academic performance (cortical preservation). We hypothesise that these findings are likely attributable to cognitive compensation strategies, such as persistent repetition, which the patient spontaneously employs, and are further supported by her other preserved cognitive functions, such as auditory working memory. Further research is essential to develop improved strategies for children with memory impairments, aiming to enhancing their daily functioning and academic performance.

**Disclosure of Interest:**

None Declared

